# Fire Ants, *Solenopsis invicta*, Dry and Store Insect Pieces for Later Use

**DOI:** 10.1673/031.008.3901

**Published:** 2008-05-19

**Authors:** Glivery G. Gayahan, Walter R. Tschinkel

**Affiliations:** Department of Biological Science, Florida State University, Tallahassee, FL 32306-4370

**Keywords:** food storage, larval feeding, dye tracers, foraging, stockpile, food drying

## Abstract

Whereas long-term storage of liquid food in the crops of worker ants and storage of dry seeds are well-known, widespread, and sometimes spectacular phenomena, there have been no previous reports documenting the storage of dead insect prey. Predacious ants typically devour their insect prey within a short time. Given a bonanza of insect prey, the fire ant, *Solenopsis invicta*, desiccates small pieces of these insects (creating insect “jerky”) and stockpiles these pieces in its mound, immediately below the mound surface, the driest and warmest location in the nest. Feeding colonies fluorescently dyed beetle larvae, and searching for fluorescence at night under ultraviolet light illumination verified such stockpiling. Stockpiles ranged from a few pieces to hundreds. Ant larvae in field colonies fed a single dose of dyed beetle larvae remained fluorescent for about two weeks. Laboratory colonies were fed a single dose of dyed larvae and then either starved of insect food, or fed on undyed larvae. All larvae in starved colonies remained strongly fluorescent for four weeks, whereas those in fed colonies gradually declined in fluorescence, showing that in the absence of an inflow of insect prey, workers in the starved colonies fed the dried insect fragments to larvae. Storage of dried food is easily overlooked, and it is possible that it is not limited to fire ants.

## Introduction

The storage of food is an obvious strategy against future shortages. Food storage occurs sporadically among animals, with the most highly developed forms occurring among humans. Among social insects, long-term, internal storage of liquid foods has evolved among ants, giving rise in the extreme case to honeypot ants such as the desert-dwelling species of the genus *Myrmecocystus* in which a replete caste stores liquid food in the crop for months (Chew 1995; Conway 1975; Conway 1977; Edwards 1873). External storage of honey and pollen in specially constructed, waxen cells, or in cells excavated in soil or wood has evolved among several species of bees. The honeybee, *Apis mellifera*, is the best known example (Winston 1987), but many species of social and nonsocial bees provide stores for their brood and/or for surviving seasons of scarcity. The high concentration of sugar in honey prevents most spoilage by microorganisms. Seeds, being alive, resist spoilage through a number of mechanisms. On the other hand, animal prey usually spoils rather rapidly *post mortem*, a fact that has led to the evolution of prey paralysis through venoms in many insects that provide insect prey for their brood, that consume the prey alive. Such behavior is widespread among the Hymenoptera, and has even been reported for the specialist ant *Cerapachys turneri* (Hölldobler 1982). The storage of solid foods, at least among ants, seems to be limited to seeds (Carroll and Risch 1984; Davidson 1977; MacKay 1981; Tschinkel 1999), which, by their very nature, invite storage. Whereas seed storage has evolved several times among the ants, there are no reports of the storage of other solid foods.

We have often noticed that when laboratory colonies of fire ants (*Solenopsis invicta*) are offered a surplus of insects to eat, they cut these insects into small pieces and deposit them in piles on top of the glass covering their nest. After years of seeing this processing behavior, it finally occurred to one of us (WRT, aka Dr. Dense) to ask whether the ants similarly stockpile dried insect prey pieces in their natural nests. We report here that indeed they do.

The fire ant, *S. invicta*, is a territorial, omnivorous exotic ant from northern Argentina that has become very abundant in disturbed habitats throughout the southeastern USA and has recently appeared in Taiwan, Hong Kong and China. Its biology was recently summarized in a book by Tschinkel (2006). It is an excellent subject for the study of many aspects of ant biology.

## Materials and Methods

Larvae of the large tenebrionid beetle, *Zophobas atratus*, which has been cultured as ant food in our laboratory for decades, were cut in half longitudinally and stained in a 0.5% solution of rhodamine B dye in water. These deep pink larvae were offered, still wet, to fire ant colonies, and were accepted readily, both in the laboratory and in the field. The pink color made it easy to distinguish the pieces of beetle larvae from soil and sand, a distinction that became especially dramatic under ultraviolet light illumination, when the rhodamine dye fluoresced a brilliant orange. Ultraviolet light also allowed us to determine which ant larvae had fed on the dyed beetle larvae, for they glowed like Chinese lanterns under this illumination.

Several field colonies were offered 10 g of dyed *Z. atratus* larvae in covered Petri dishes with holes in the sides. After 24 hours, the reduction of the weight of the larvae was measured to determine how much larval material the colonies had collected; most collected more than half. One day after such feeding, chloroform was sprinkled over the mounds to anaesthetize the ants, and the mounds and the nests below them were dissected at night under ultraviolet illumination. The pieces of dyed larvae were easily seen under these conditions.

In the laboratory, fire ant colonies were maintained in dampened, soil-free plaster nests at about 28° C, and fed sugar water and beetle larvae. The nests were kept in photographic trays whose sides had been painted with fluon to prevent escape.

## Results

**Figure 1.**
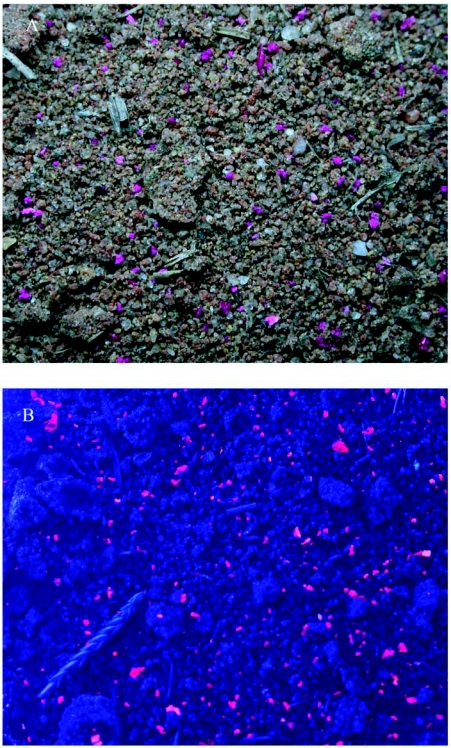
(A) Soil taken from the mound of a fire ant colony fed rhodamine-dyed beetle larvae contains localized concentrations of hundreds of pieces of the dyed larvae. (B) The pieces fluoresce brightly under ultraviolet light

**Figure 2.**
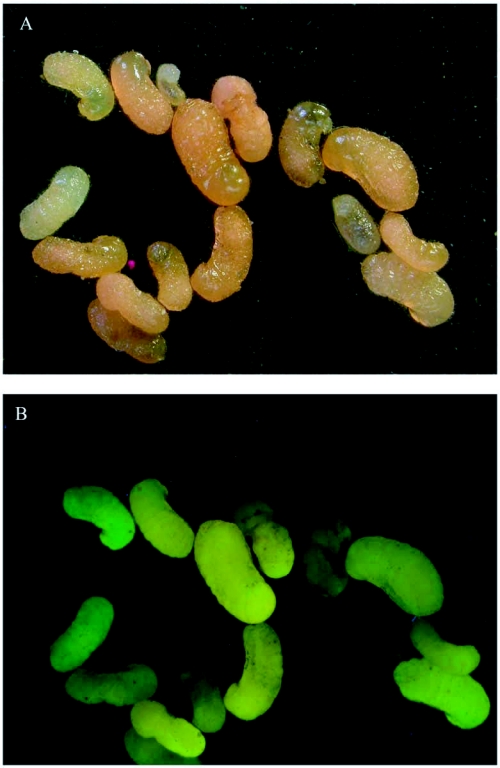
(A) Fire ant larvae that have eaten dyed beetle tissue are pink under visible light and (B) fluoresce bright yellow-green to orange under ultraviolet light.

Preliminary experiments in which field colonies were fed dyed *Z. atratus* larvae showed that small dirt samples scooped from the mound often contained pink bits that fluoresced strongly under ultraviolet light. Sometimes such mound samples contained hundred of pieces in a small area of the mound, suggesting that the ants aggregated the pieces there (Figure 1). Moreover, larvae taken from such colonies were pink and also fluoresced under ultraviolet light, showing that they had fed on the dyed *Z. atratus* tissue (Figure 2).

**Figure 3.**
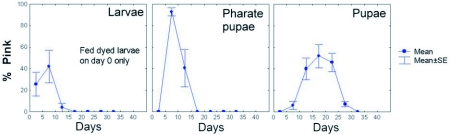
Feeding colonies with dyed beetle larvae for 24 hours on day zero results in a pulse of dyed brood that passes through development in about a month. The peak in dyed larvae and pharate pupae after about a week (rather than on the first day) suggests that stored beetle pieces are fed to ant larvae over at least a week.

A set of five field colonies were fed wet, dyed beetle larvae for 24 hours only and their brood sampled at intervals for one month. The dye appeared first in the feeding larvae and pharate pupae, peaking after about a week (Figure 3). This was followed by a peak in the number of dyed pupae at about 17 days, at which time no dyed ant larvae or pharate pupae were present any longer, showing that the rhodamine dye had labeled a pulse of brood. The fact that the peak in larval and pharate pupal color did not occur on the first day after feeding suggests that the stockpiled, dyed pieces were fed to the larvae over a period of at least a week.

Observations of workers collecting material from the wet, dyed larvae showed that they were not merely cutting pieces for transport, but were first sucking as much liquid from the tissue as possible. We have also observed this behavior when baiting ants with tuna--- the tuna is first broken up into smaller pieces which are then carried back to the nest. Presumably, the fluid from the prey is returned to the nest in the crops of workers, where it can be shared by trophallaxis. In any case, the prey pieces, be they tuna or beetle larvae are processed into what can fairly be labeled “insect jerky” before transport.

**Figure 4.**
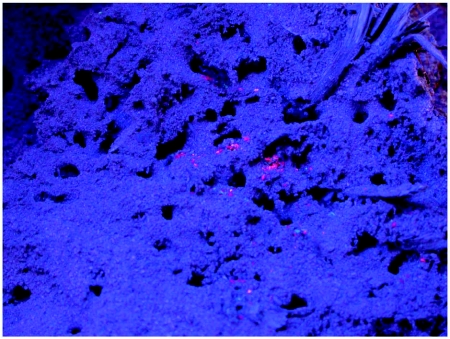
With only the surface of the mound removed during a nocturnal excavation, ultraviolet light revealed the location of caches of dyed beetle “jerky”. Note that the jerky lies on the floor of the galleries, and is not incorporated into the mound structure.

**Figure 5.**
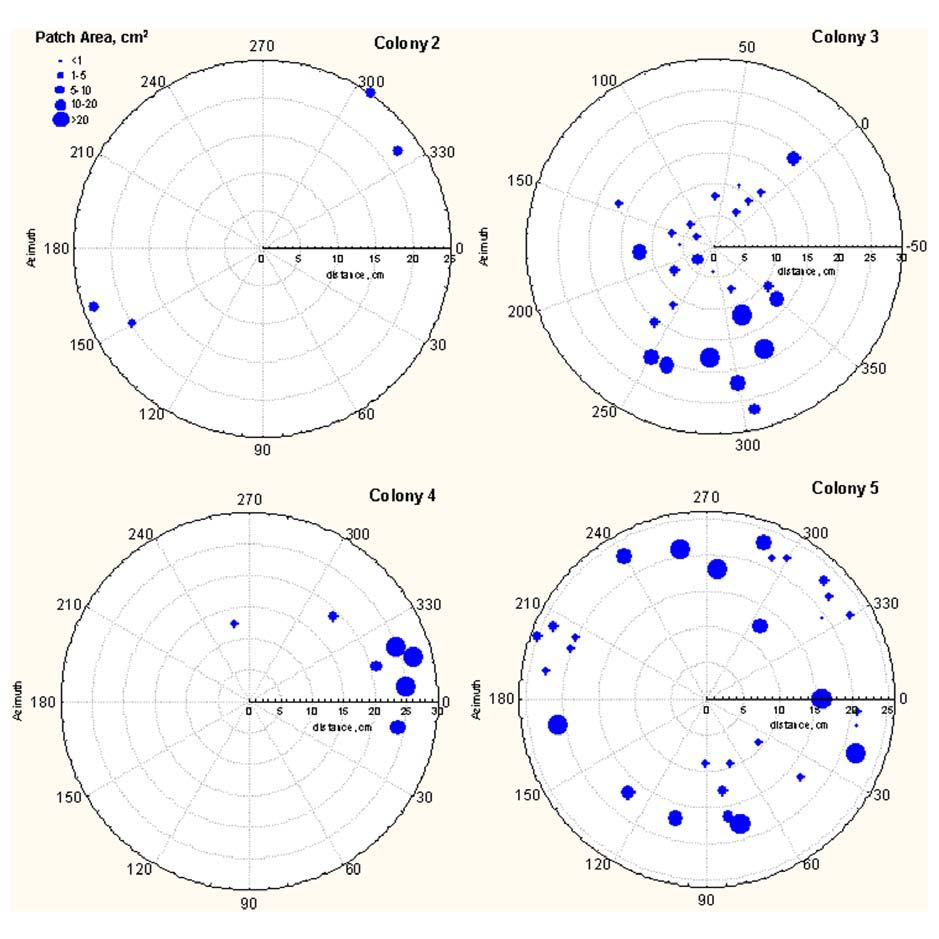
Nocturnal dissection of the mounds of four fire ant colonies, showing the location and size of the clusters of fluorescent beetle larva pieces. Each circular graph represents the approximate outline of the mound, seen from above. All piles were located immediately under the surface of the mounds, the warmest and driest location in the nest.

A more careful analysis of the location of stockpiles in the nest was carried out by dissecting four chloroformed fire ant colonies at night under ultraviolet light (Figure 4). We mapped the location and size of fluorescent stockpiles by placing a transparent plastic sheet over the mound and outlining the piles. All piles were located immediately under the mound surface, the driest and warmest location in the nest (Figure 5). Only a single pile was located about 2 cm below the surface (not shown in Figure 5). Upon reaching the level at which larvae were kept in each of the four nests, we found the brood to be strongly fluorescent, indicating that they had been fed the dyed pieces of *Z. atratus*. A fifth colony was excavated a week after feeding, at which time, only two small piles of fluorescent pieces were found. This suggests that the ants are not simply incorporating the pieces into the mound as though they were soil, but are storing them for eventual feeding to the ant larva0e. This was later verified in the laboratory experiment described below.

To study the consumption of insect jerky, we set up four laboratory colonies in soil-free nests and fed them dyed *Z. atratus* larvae for 24 hours. Two colonies were fed no insect food whatsoever after the first 24 hours (starved group), and the other two were fed undyed *Z. atratus* larvae *ad libitum* (fed group). All colonies were given sugar water *ad libitum*, but for the starved group, the only source of insect tissue for feeding 4^th^ instar ant larvae was the dyed insect jerky. The intensity and percentage of dye in brood was measured for about a month.

**Figure 6.**
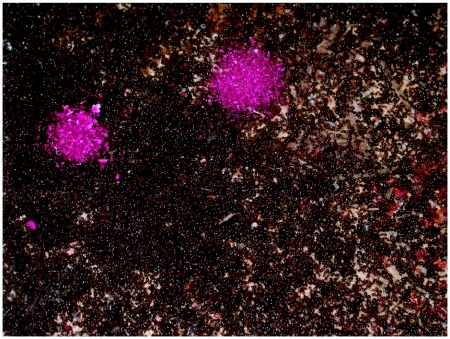
Two piles of dyed “insect jerky” on top of the glass cover of a laboratory nest. Note the pink brood on the right side of this image.

**Figure 7.**
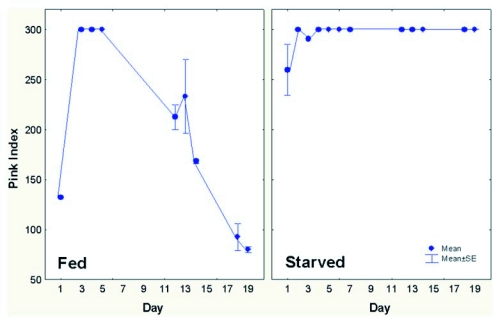
Dye in feeding larvae gradually declined in the fed group, as undyed jerky gradually diluted the dyed. In the starved group, the only jerky available was the dyed store, so that the feeding larvae continued to be maximally dyed.

All the experimental colonies converted the dyed *Z. atratus* larvae into jerky and piled these bits on top of the glass nest cover (Figure 6). Feeding larvae became intensely pink and fluorescent within 24 hr, and as in the field experiment, the dye appeared in pupae a few days later. Dye intensity in the brood was estimated as strong, moderate, or weak. These estimates were assigned values of 3, 2 and 1, respectively, and were multiplied by the percent of brood that were perceptibly dyed to produce a “dye index” whose values ranged from 0 to 300. Over a period of 19 days, the dye index of larvae in the fed group declined from 300 to about 80, whereas that of the starved group remained at about 300 for the duration of the experiment (Figure 7). Undyed jerky gradually diluted the dyed jerky in the fed group, but no such dilution occurred in the starved. Moreover, the total area of the jerky piles decreased by about 50% in the fed group, but about 95% in the starved.

These results showed that the workers actually feed the jerky to their larvae over an extended period, in other words, they retrieve this food from storage.

## Discussion

Humans dry many different kinds of food, including the flesh of animals, for long-term storage, but until now, no one has reported that ants do so as well. Perhaps this ant behavior has not been reported previously as it is hard to detect because the pieces of insects look like soil grains. It is thus possible that this behavior is fairly widespread among ants, but it is also possible that it is limited to ants whose nests include very dry chambers. The rainy tropics would thus seem unlikely places where ants would store dried insects, but deserts, or ants that build rapidlydrying mounds would be better suited. No doubt, it would require very careful excavation to detect such storage, and the drier the soil, the more likely is collapse and obfuscation during excavation. Feeding dyed insect prey was found to be a good way to get around these problems, for the jerky would be detectable under ultraviolet light.

The fact that no fluorescent bits were detected between just beneath the mound surface and the larval areas suggests that workers retrieve the jerky bits from storage and bring them directly to the larvae, without intermediate stockpiling. Rain would seem to be a challenge to storage, for the jerky would become wet and subject to spoilage. Perhaps the mound usually dries fast enough to prevent such spoilage.

The evolution of this behavior may be associated with the different manner in which ants collect and transport liquid and solid food. Liquids are taken into the distensible crop and shared by trophallaxis, whereas solids are transported in the mandibles and fed to the 4^th^ (final) instar larvae, the only larval instar that eats solid food (Petralia and Vinson 1978). Workers place a food pellet on the ventrum of the 4^th^ instar larva in a special “basket” of inward pointing hairs. The larva covers the pellet with salivary secretion, which partly converts it into a liquefied product, which is then collected and distributed around the colony by worker trophallaxis (Cassill and Tschinkel 1999). This tendency to separate the liquid food stream from the solid may underlie the evolution of the preparation of jerky.
